# Microbial Dysbiosis Is Associated with Human Breast Cancer

**DOI:** 10.1371/journal.pone.0083744

**Published:** 2014-01-08

**Authors:** Caiyun Xuan, Jaime M. Shamonki, Alice Chung, Maggie L. DiNome, Maureen Chung, Peter A. Sieling, Delphine J. Lee

**Affiliations:** 1 Dirks/Dougherty Laboratory for Cancer Research, Department of Translational Immunology, John Wayne Cancer Institute, Santa Monica, California, United States of America; 2 Pathology Department, Saint John’s Health Center, Santa Monica, California, United States of America; 3 Department of Medicine, Cedars-Sinai Medical Center, Los Angeles, California, United States of America; 4 Margie Petersen Breast Center, Saint John’s Health Center, Santa Monica, California, United States of America; Virginia Commonwealth University School of Medicine, United States of America

## Abstract

Breast cancer affects one in eight women in their lifetime. Though diet, age and genetic predisposition are established risk factors, the majority of breast cancers have unknown etiology. The human microbiota refers to the collection of microbes inhabiting the human body. Imbalance in microbial communities, or microbial dysbiosis, has been implicated in various human diseases including obesity, diabetes, and colon cancer. Therefore, we investigated the potential role of microbiota in breast cancer by next-generation sequencing using breast tumor tissue and paired normal adjacent tissue from the same patient. In a qualitative survey of the breast microbiota DNA, we found that the bacterium *Methylobacterium radiotolerans* is relatively enriched in tumor tissue, while the bacterium *Sphingomonas yanoikuyae* is relatively enriched in paired normal tissue. The relative abundances of these two bacterial species were inversely correlated in paired normal breast tissue but not in tumor tissue, indicating that dysbiosis is associated with breast cancer. Furthermore, the total bacterial DNA load was reduced in tumor versus paired normal and healthy breast tissue as determined by quantitative PCR. Interestingly, bacterial DNA load correlated inversely with advanced disease, a finding that could have broad implications in diagnosis and staging of breast cancer. Lastly, we observed lower basal levels of antibacterial response gene expression in tumor versus healthy breast tissue. Taken together, these data indicate that microbial DNA is present in the breast and that bacteria or their components may influence the local immune microenvironment. Our findings suggest a previously unrecognized link between dysbiosis and breast cancer which has potential diagnostic and therapeutic implications.

## Introduction

One in eight women will be diagnosed with breast cancer in their lifetime. It is the second leading cause of death in women, with >40,000 deaths annually [1]. Despite the $5.5 billion spent on breast cancer research over the past twenty years, the origins of a majority of breast cancer cases (estimated to be as high as 70%) remain unknown [2]. It is crucial to understand how these sporadic breast cancers arise in order to develop preventative strategies against this devastating disease. The recent appreciation of the influence of microbiota on human health and disease begs the question of whether microbes play a role in sporadic breast cancers of unknown etiology.

Microbes inhabiting the human body outnumber human cells 10:1. Their influence on human health and disease is a new and rapidly expanding area of research. Microbes have been linked to diseases as varied as obesity [3], [4], colon cancer [5], [6] and colitis [7]. In obese individuals, the ratio of Firmicutes to Bacteroidetes in the colon is significantly higher than in lean individuals [3], [8]. Placing obese individuals on low-fat diets resulted in a decrease in this ratio, though not to the levels seen in lean individuals [8]. In colon cancer, the overabundance of a single bacterial species *Fusobacterium nucleatum* correlates with disease and increased likelihood of lymph node metastasis [6]. In contrast to the pathogenic nature of *Fusobacterium* in colon cancer, the bacterium *Bacteroidetes fragilis* exerts a protective effect against colitis by modulating inflammatory immune responses in the gut [9]. From these and other recent studies, it is becoming increasingly apparent that both community composition and discrete bacterial species can exert either pathogenic effects that encourage disease development or probiotic effects that maintain health status.

Previous studies of microbial causes of breast cancer have focused on specific viruses and their potential contributions to breast cancer. While HPV infection has been reported by some groups to be associated with breast cancer [10], [11], [12], others have failed to find a correlation [13], [14]. Similarly, some groups have reported that up to 50% of breast tumors are EBV-positive [15], [16], [17], [18], while others have been unable to detect the virus in breast tumors altogether [19], [20]. In contrast to viruses, bacteria in the breast have been studied to a far lesser extent. Several groups have investigated the bacteria responsible for infections stemming from breast implant procedures using culture-based methods [21]. Further, the breast milk of healthy women has been shown to harbor an abundance of bacterial species including commonly found skin bacteria [22], [23]. Bacteria in the breast have been studied in the context of infections and in healthy individuals, but no comprehensive study of bacteria in breast cancer has been reported. Here, we characterized and compared the microbiota in breast tumor and paired normal tissue and identified dysbiosis that was associated with breast cancer disease state and severity.

## Materials and Methods

### Ethics statement for obtaining breast tissue specimens

Formalin fixed paraffin-embedded (FFPE) and fresh-frozen breast tissues were obtained from Saint John’s Health Center in accordance with institutional IRB requirements approved by the Saint John's Health Center/John Wayne Cancer Institute joint institutional review board and Western institutional review board (Western IRB). Written consent was specifically waived by the approving IRB.

### Genomic DNA (gDNA) extraction from FFPE tissue

Total genomic DNA was extracted from FFPE breast tissues using QIAamp DNA FFPE Tissue kit per manufacturer’s instructions with slight modifications. Purified gDNA was eluted twice from the column using ultrapure water. All extractions were performed in a designated clean (pre-PCR) room.

### 16S pyrosequencing

We initially set out to investigate the microbiome in breast cancer, and elected to study ER+ tumors. Due to the variability of the microbiome from individual to individual, we decided matched tissue (paired normal and tumor) from the same individual would provide the best comparison of microbial communities. Twenty paraffin-embedded paired samples were used for this purpose. Genomic DNA (gDNA) (from Subjects 1–20) was submitted to Second Genome Inc., for pyrosequencing and analysis. The gDNA was amplified using fusion primers targeting the bacterial 16S V4 rDNA with indexing barcodes. All samples were amplified with two differently barcoded V4 fusion primers and pooled for sequencing on the Illumina Miseq with 150bp paired-end reads. 60,248±14,229 (mean ± s.d.) reads were obtained per sample. Sequences were quality filtered and demultiplexed using QIIME [24] and custom scripts with exact matches to the supplied DNA barcodes. Resulting sequences were then searched against the Greengenes reference database of 16S sequences [25] and clustered at 97% by UCLUST [26]. The longest sequence from each OTU was used as the OTU representative sequence and assigned taxonomic classification via Mothur’s Bayesian classifier [27] and trained against the Greengenes database clustered at 98%. To account for biases caused by uneven sequencing depth, equal numbers of random sequences were selected from each sample prior to calculating community-wide dissimilarity measures. The sequence data has been submitted to the European Nucleotide Archive, PRJEB4755.

### Quantitative PCR (qPCR) for bacterial copy numbers

qPCR was performed using universal bacterial rDNA primers 63F (forward, 5′-GCA GGC CTA ACA CAT GCA AGT C-3′) and 355R (reverse, 5′-CTG CTG CCT CCC GTA GGA GT-3′) on microbial DNA extracted from FFPE tissue. All samples from pyrosequencing were also assessed for bacterial copy number (Subjects 1-20, excluding Subjects 3 and 5 due to limited material) and additional paraffin-embedded tissue specimens (from patients with breast cancer-subjects 21–41) were obtained at a later time after the initial pyrosequencing experiment, and thus were used only in the quantification experiments as previously described [28] to enumerate the amount of total bacteria. DNA from healthy specimens was obtained from patients undergoing reduction mammoplasty, with no evidence of breast cancer. Bacterial copy numbers were normalized by the total amount (µg) of extracted DNA quantified using Quanti-it PicoGreen dsDNA Reagent Kit (Invitrogen). Samples were randomized and processed in a blinded manner. The genus-specific primers Sph-spt 694F (forward, 5′-GAG ATC GTC CGC TTC CGC-3′) and Sph-spt 983R (reverse, 5′-CCG ACC GAT TTG GAG AAG-3′) were used to quantify Sphingomonas [29]. The species-specific primers 5F (forward, 5′- CTT GAG TAT GGT AGA GGT T-3′) and 8R (reverse, 5′-CAA ATC TCT CTG GGT AAC A-3′) were used to quantify *M. radiotolerans* [30] (Subjects 1–20).

### qPCR array

Given the superior quality of mRNA from fresh-frozen tissue, we elected to use fresh-frozen tissue rather than formalin fixed, paraffin embedded tissue in our gene expression study. RNA was extracted from fresh-frozen breast tissue from three healthy reduction mammoplasty patients and from tumor tissue of six patients with breast cancer (Subjects 42–47), then converted to cDNA using iScript cDNA synthesis kit (Biorad). cDNA was added to Human Antibacterial Response PCR Arrays (Qiagen) and the arrays were processed according to manufacturer’s instructions. Data were analyzed using RT^2^ Profiler PCR Array Data Analysis Software version 3.5, using beta-actin gene expression for normalization.

### Statistical analysis

Student’s t tests, Kruskal-Wallis nonparametric ANOVA tests and Spearman correlation tests were performed using Graphpad Prism software (Graphpad). Cuzick’s Trend tests were performed using StatsDirect statistical software (StatsDirect). p<0.05 was used as the cut-off value for statistical significance.

## Results

### Survey of the breast microbiota in breast cancer patients

**Figure 1:**
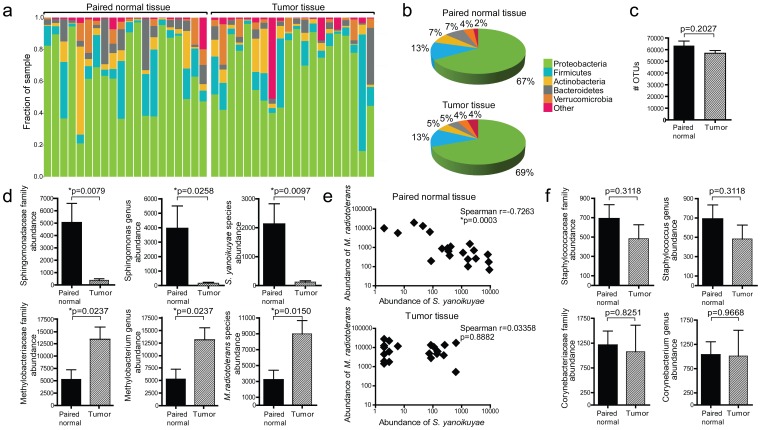
Survey of microbial communities residing in breast tissue from breast cancer patients. **A**) Phylum level distribution of microbial communities comparing paired normal adjacent (“paired normal”) and breast cancer tissue from 20 patients with ER-positive breast cancer (n = 20). Each bar represents 100% of the bacteria detected in a given sample. **B**) Combined distribution at the phylum level in paired normal and breast tumor tissue (n = 20). **C**) Number of OTUs found in each community (n = 20). **D**) Analysis of OTUs with differential abundance between paired normal and tumor tissue (n = 20). **E**) Correlation of relative abundances of *M. radiotolerans* and *S. yanoikuyae* (n = 20). **F**) Relative abundances of commonly found skin bacteria (n = 20). p-values from Student’s paired t-test are shown, with P<0.05 considered significant. Error bars represent mean ± s.e.m.

The breast cancer microbiome has thus far not been described. We surveyed the breast microbiota in paired normal adjacent tissue (“paired normal”) and tumor tissue from 20 patients with estrogen receptor (ER)-positive breast cancer (clinical data reported in Table S1) using 16S pyrosequencing. Across the samples, the five richest phyla consisting of Proteobacteria, Firmicutes, Actinobacteria, Bacteroidetes and Verrucomicrobia, accounted for an average of 96.6% of all sequences across samples (Figure 1a, b). No clustering of samples was observed on the basis of histopathology or tumor stage using principle coordinates analysis (PCoA) (Figure S1). The number of operational taxonomic units (OTUs) detected did not vary between paired normal and tumor tissue, indicating that there was no significant difference in richness between the sampled communities (Figure 1c). However, the evenness of the communities was significantly different (Adonis testing, p = 0.01). Of the 1614 OTUs detected, 11 OTUs were differentially abundant (p<0.05, Table S2). Of those 11 OTUs, eight were more abundant in paired normal tissue and three were more abundant in tumor tissue. 50% (4/8) of the OTUs identified as more abundant in paired normal tissue belonged to the genus Sphingomonas and 66.7% (2/3) of the OTUs identified as more abundant in tumor tissue belonged to the genus Methylobacterium (Table S2). The bacterium *Sphingomonas yanoikuyae* (*S. yanoikuyae*) was the most significantly enriched (p = 0.0097, Figure 1d, top panel) and the most prevalent (found in 95% of samples) in paired normal tissue (Table S2). The bacterium *Methylobacterium radiotolerans* (*M. radiotolerans*) was the most significantly enriched (p = 0.0150, Figure 1d, bottom panel) and the most prevalent (found in 100% of samples) in tumor tissue. In contrast, the relative abundances of common skin bacteria including Staphylococcus and Corynebacterium did not vary significantly between paired normal and tumor tissue (Figure 1f). Since pyrosequencing provides a qualitative survey of relative abundances of microbiota, we used qPCR to determine if there was a quantitative difference in the levels of *S. yanoikuyae* and *M. radiotolerans* in paired normal and tumor tissue. Sphingomonas was detected in 40% of paired normal tissue and none of the corresponding tumor tissue, with absolute levels of Sphingomonas being significantly higher in paired normal tissue (p = 0.0363, Figure S2). In contrast, though *M. radiotolerans* was detected in all samples by qPCR, its absolute levels did not vary significantly between paired normal and tumor tissue (p = 0.2508, Figure S2), indicating that its higher relative abundance in tumor tissue reflects a decrease in other bacteria present and not an increase in the absolute level of the organism. Interestingly, there was a strong inverse correlation between the abundance of *S. yanoikuyae* and *M. radiotolerans* in paired normal tissue (Figure 1e top panel, p = 0.0003) which was not found in the corresponding tumor tissue (Figure 1e bottom panel). These data suggest that in paired normal tissue, *S. yanoikuyae* and *M. radiotolerans* may occupy similar niches and thus counterbalance each other in abundance. Meanwhile in tumor tissue, the quantity of *S. yanoikuyae* becomes significantly lower as the quantity of *M. radiotolerans* remains constant.

### Reduction in bacterial load in advanced stage breast tumors

**Figure 2:**
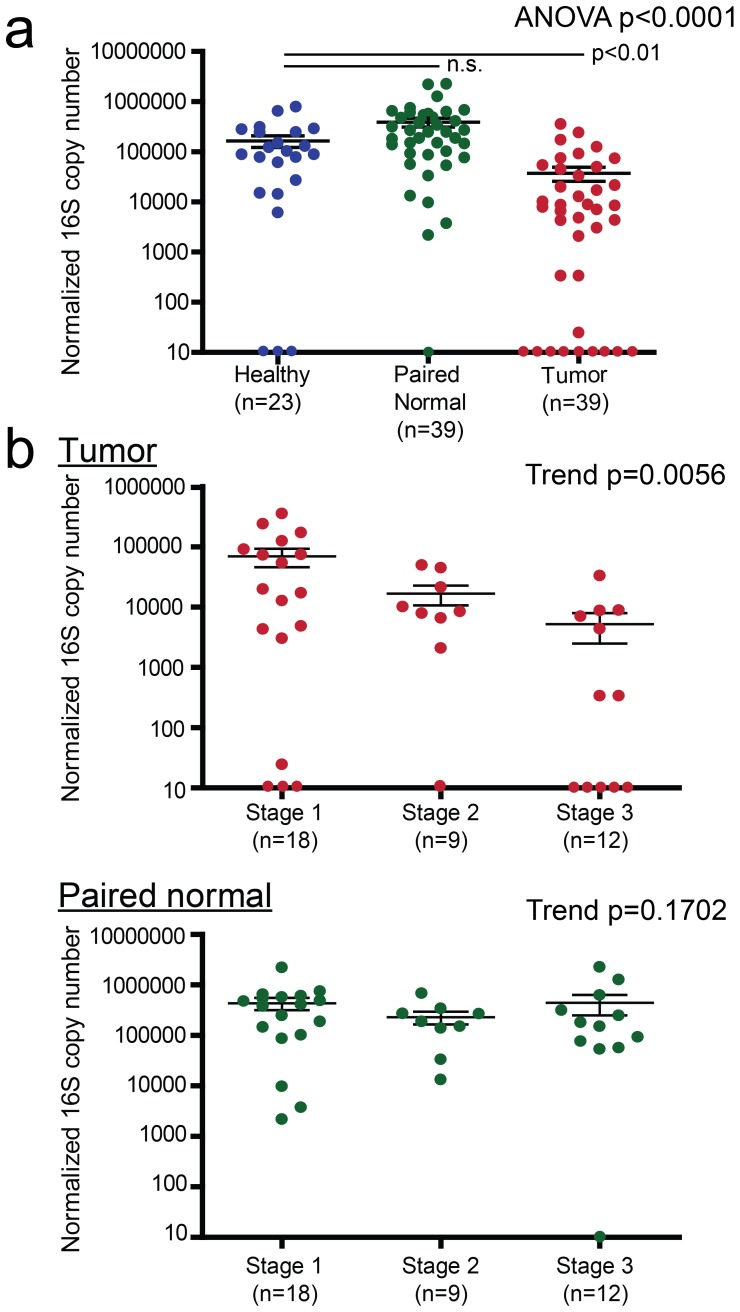
Quantification of bacterial load in tissue from healthy and breast cancer patients. **A**) Copy numbers of the bacterial 16S gene were compared among healthy (age-matched) (n = 23), paired normal (n = 39) and tumor tissue (n = 39). Healthy specimens were obtained from patients undergoing reduction mammoplasty, with no evidence of breast cancer. Statistical analysis was performed using Kruskal-Wallis nonparametric ANOVA with Dunn’s Multiple Comparison post-test. **B**) Bacterial load in tissue according to clinical staging of the tumor specimen. Statistical analysis was performed using Cuzick’s Trend test. All statistical analyses were considered significant when P<0.05. Data represent the average of duplicate values. Error bars represent mean ± s.e.m.

To further explore quantitative differences in the microbiota, qPCR analysis was performed to enumerate 16S ribosomal DNA (rDNA) copy numbers as a surrogate measure of total bacterial counts [28]. Results showed 10-fold more bacteria in tumor tissue (37,582±11,783) compared to paired normal tissue (391,096 ±81,570), while bacterial levels in paired normal tissue did not differ significantly from those found in healthy breast tissue (164,484±42,477) (mean ± s.e.m.) using Kruskal-Wallis nonparametric ANOVA with Dunn’s Multiple Comparison post-test to account for uneven sample numbers between the three groups studied (healthy vs. tumor p<0.01, paired normal vs. tumor p<0.001, healthy vs. paired normal n.s., Figure 2a). Moreover, an inverse correlation between breast cancer stage and bacterial load in tumor tissue, but not in paired normal tissue, was observed using Cuzick’s Trend test analysis (Figure 2b). Tumors from Stage 1 patients had the highest copy numbers of bacterial DNA (69,489±23,382) (mean ± s.e.m.), followed by Stage 2 patients (16,867±6,152), with Stage 3 patients having the lowest bacterial load amongst the three groups (5,258±2,758) (Trend p = 0.0056) (Figure 2b, top panel). In contrast, paired normal tissue from the same patients did not have different bacterial copy numbers (Trend p = 0.1702) (Figure 2b, bottom panel). These data suggest an inverse correlation between severity of disease and bacterial load at the tumor site, which may have diagnostic implications in breast cancer.

### Reduction in expression of antibacterial response genes in breast tumors

**Figure 3:**
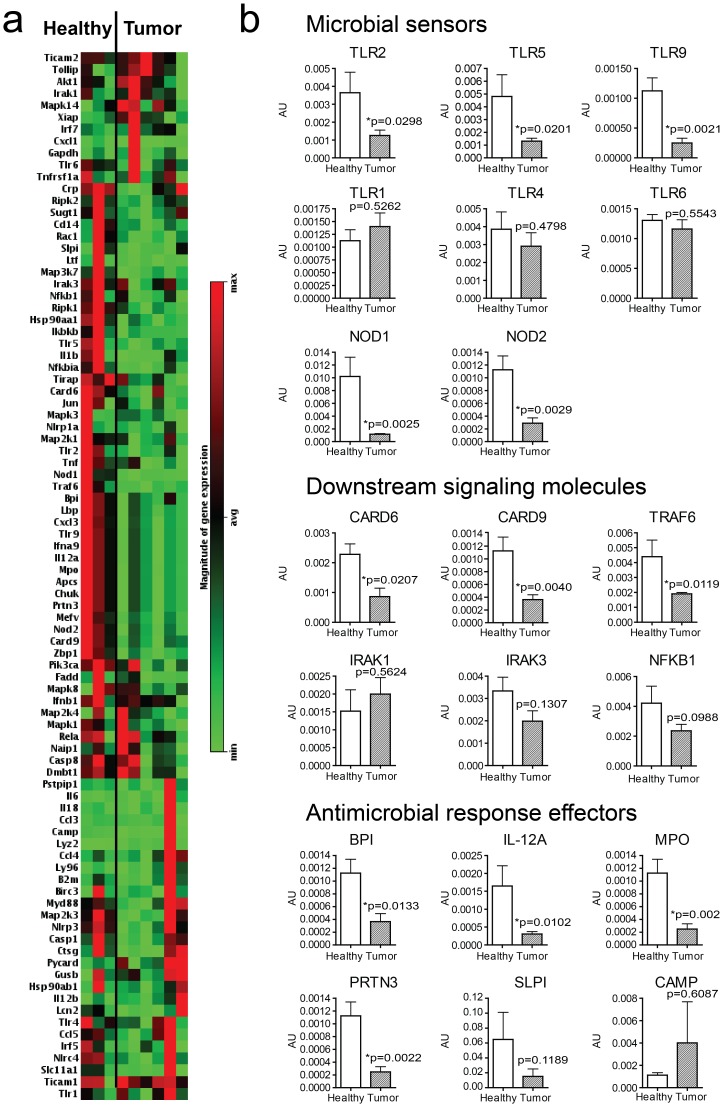
Expression profiles of antibacterial response genes in healthy and breast cancer tissue (n = 9). Healthy specimens were obtained from patients undergoing reduction mammoplasty, with no evidence of breast cancer. **A**) Heatmap of gene expression values generated using non-supervised hierarchical clustering **B**) Expression profiles of antimicrobial response genes. p-values from Student’s paired t-test are shown, with p<0.05 considered significant. Error bars represent mean ± s.e.m.

We hypothesized that the decreased bacterial load measured in tumor tissue compared with paired normal tissue and healthy tissue may influence the expression of antibacterial response genes in the tumor microenvironment. To test this hypothesis, we compared gene expression profiles in breast tissue from three healthy patients undergoing reduction mammoplasty and six patients with breast cancer (tumor tissue was used, clinical data reported in Table S1) using a targeted gene array for human antibacterial response genes normalized to the housekeeping gene beta-actin. One-third (28/84) of antibacterial genes surveyed were downregulated in tumor tissue, while the remaining two-thirds (56/84) were not significantly different between tumor and healthy tissue. Strikingly, none of the antibacterial genes surveyed were significantly upregulated in tumor tissue. We found that the samples segregated into their tissue type, tumor vs. healthy by non-supervised hierarchical clustering and a subset of genes were comparatively decreased in expression in tumor tissue compared with healthy tissue (Figure 3a). Of these genes, the transcripts of microbial sensors Toll-like receptors 2, 5 and 9 (TLR2, TLR5 and TLR9) were significantly reduced in tumor tissue (p = 0.0298, p = 0.0201 and p = 0.0021, respectively), while expression levels of Toll-like receptors 1, 4 and 6 (TLR1, TLR4 and TLR6) were similar in healthy and tumor tissue (Figure 3b). *S. yanoikuyae* is a species of Gram-negative bacteria that does not contain lipopolysaccharide (LPS) and therefore does not elicit TLR4-mediated responses [31]. The cytoplasmic microbial sensors NOD receptors 1 and 2 (NOD1 and NOD2) were also expressed at lower levels in tumor tissues (p = 0.0025 and p = 0.0029, respectively), along with downstream signaling molecules for innate microbial sensors including CARD6, CARD9 and TRAF6 (p = 0.0207, p = 0.0040 and p = 0.0119, respectively) (Figure 3b). In addition, transcripts of antimicrobial response effectors were less abundant in tumor tissue, with BPI, MPO and PRTN3 levels being significantly lower compared with those found in healthy tissue (p = 0.0133, p = 0.002 and p = 0.0022, respectively) (Figure 3b). These data show a significant reduction in antibacterial responses in breast cancer tumor tissue. Whether breast microbiota can influence the local immune microenvironment of the breast requires further study.

## Discussion

Since traditional culture-based methods tend to underestimate and bias the microbial diversity in a given sample, the role of microbes in breast carcinogenesis has not been thoroughly explored. Here, we used next-generation sequencing techniques to perform a high-resolution survey of the resident breast microbiota in tumor and paired normal breast tissue from breast cancer patients. In addition, we investigated a potential association of bacterial load with levels of immune gene expression by quantifying the amount of bacteria present in healthy and tumor tissue and correlating bacterial load with the magnitude of antibacterial immune responses in the tissue.

Previous paradigms of microbes in disease point to specific pathogenic bacteria as exclusive causes. Indeed, *Helicobacter pylori* infection is known to promote gastric cancer and gastric mucosal-associated lymphoid tissue (MALT) lymphoma [32], [33]. Reports have also linked the presence of pathogenic *Escherichia coli* containing *pks* toxicity genes with local tissue inflammation and colon carcinogenesis [34]. However, recent studies have revealed that the interactions between bacteria and host can be far more complex. First, microbial community composition and relative abundance of bacterial species can be contributory factors to health and disease [3], [4], [35]. Second, not all bacteria are pathogenic; in fact, some bacteria have probiotic effects that help to maintain health status [9]. An example of this is the bacterium *Bacteroidetes fragilis*, a probiotic organism that protects against experimental colitis by suppressing production of the proinflammatory cytokine IL-17 in the gut [7], [9]. As in the gut, the presence of a specific bacterium may be beneficial in the breast. In our study, the association of *S. yanoikuyae* with normal breast tissue and the dramatic reduction in its abundance in corresponding tumor tissue suggests that this organism may have probiotic functions in the breast. Interestingly, *S. yanoikuyae* express glycosphingolipid ligands, which are potent activators of invariant NKT (iNKT) cells [31]. iNKTs are important mediators of cancer immunosurveillance [36] and have been reported to have an integral role in controlling breast cancer metastasis [37]. Further studies are aimed at investigating the potential role of *S. yanoikuyae* in breast cancer development and progression.

In a quantitative survey of breast microbiota, we found that the amount of bacteria was not significantly different in paired normal tissue from breast cancer patients and healthy breast tissue from healthy individuals. However, compared to both these tissues, breast tumor tissue had significantly reduced amounts of bacteria. This reduction coincided with reduced expression of one-third of antibacterial response genes surveyed. Innate immune sensors including TLR 2, 5 and 9 and antimicrobial response effectors IL-12A, BPI and MPO were all expressed at lower levels in tumors compared to healthy breast tissue. Taken together, these data suggest that bacteria may have a role in maintaining healthy breast tissue through stimulation of host inflammatory responses.

The notion that bacteria can provide protective immune stimulation against disease has been put forth in several studies. The skin bacterium *Staphylococcus epidermis* has been shown to be essential in maintenance of protective immunity against the pathogen *Leishmania major* via stimulation of skin resident T cells [38]. There are also indications that bacteria may play a protective role in breast cancer. In a mouse model of sporadic breast cancer, treatment of mice with antibiotic regimens leads to increased risk of tumor development as well as higher rates of tumor growth [39]. Similarly, a study by Velicer et al. provides clinical data to support the idea that bacteria may play a role in preventing breast tumorigenesis in humans [40]. In this case-control study, increasing cumulative days of antibiotic usage correlated with increased risk of developing and succumbing to breast cancer [40]. Collectively, our data and previous reports support a model in which bacteria contribute to maintenance of healthy breast tissue by stimulating resident immune cells. When dysbiosis occurs, a reduction in the overall number of bacteria and/or the abundance of specific species such as *S. yanoikuyae*, may lead to decreased bacterial-dependent immune cell stimulation, ultimately resulting in a permissive environment for breast tumorigenesis.

The dramatic reduction in bacterial load found in breast tumor compared to healthy breast tissue warrants further study to determine if bacterial load could be an additional indicator of diagnosis or staging of breast cancer. In addition, the inverse correlation between bacterial load and tumor stage implies that bacterial load might be used in conjunction with current methods to monitor the progression of breast cancer and to facilitate staging of the disease. Furthermore, it is tempting to speculate that a decrease in bacterial load in a healthy individual may be a signal of heightened breast cancer risk. Based on our studies, further investigation into the role of microbes in breast cancer would be of interest.

## Supporting Information

Figure S1 Principle coordinates analysis (PCoA) plots of samples categorized based on histopathology (left panel, n = 20 paired samples) or tumor stage (right panel, n = 20 tumor only). No clustering based on these categories was found among samples.
(TIF)

Figure S2 Detection of Sphingomonas and *M. radiotolerans* in paired normal and breast tumor tissues (n = 20). Data represent the average of duplicate values. Data were normalized to expression levels of beta-actin. p-values from Student’s paired t-test are shown, with p<0.05 considered significant. Error bars represent mean ± s.e.m.
(TIF)

Table S1 Summary of clinical data for the breast cancer patients used in this study.
(TIF)

Table S2 OTUs enriched in paired normal or tumor tissue. Prevalence refers to the number of samples in which the indicated OTU was detectable. Paired Student’s t-tests were used to determine differences in abundances of OTUs. n.d., not detectable.
(TIF)

## References

[1] Jemal A, Siegel R, Xu J, Ward E (2010) Cancer statistics. CA Cancer J Clin 60: 277–300. 20610543 10.3322/caac.20073

[2] Madigan MP, Ziegler RG, Benichou J, Byrne C, Hoover RN (1995) Proportion of breast cancer cases in the United States explained by well-established risk factors. J Natl Cancer Inst 87: 1681–1685. 7473816 10.1093/jnci/87.22.1681

[3] Turnbaugh PJ, Ley RE, Mahowald MA, Magrini V, Mardis ER, et al (2006) An obesity-associated gut microbiome with increased capacity for energy harvest. Nature 444: 1027–1131. 17183312 10.1038/nature05414

[4] Turnbaugh PJ, Hamady M, Yatsunenko T, Cantarel BL, Duncan A, et al (2009) A core gut microbiome in obese and lean twins. Nature 457: 480–484. 19043404 10.1038/nature07540 PMC2677729

[5] Kostic AD, Gevers D, Pedamallu CS, Michaud M, Duke F, et al (2011) Genomic analysis identifies association of Fusobacterium with colorectal carcinoma. Genome Research 22: 292–298. 22009990 10.1101/gr.126573.111 PMC3266036

[6] Castellarin M, Warren R, Freeman JD, Dreolini L, Krzywinski M, et al (2012) Fusobacterium nucleatum infection is prevalent in human colorectal carcinoma. Genome Research 22: 299–306. 22009989 10.1101/gr.126516.111 PMC3266037

[7] Mazmanian SK (2008) Capsular polysaccharides of symbiotic bacteria modulate immune responses during experimental colitis. J Pediatric Gastroenterol Nutr 46: E12–12. 10.1097/01.mpg.0000313824.70971.a7 18354314

[8] Ley RE, Turnbaugh PJ, Klein S, Gordon JI (2006) Microbial ecology: Human gut microbes associated with obesity. Nature 444: 1022–1023. 17183309 10.1038/4441022a

[9] Mazmanian SK, Round JL, Kasper DL (2008) A microbial symbiosis factor prevents intestinal inflammatory disease. Nature 453: 620–625. 18509436 10.1038/nature07008

[10] Akil N, Yasmeen A, Kassab A, Ghabreau L, Darnel AD, et al (2008) High-risk human papillomavirus infections in breast cancer in Syrian women and their association with Id-1 expression: a tissue microarray study. Br J Cancer 99: 404–407. 18648363 10.1038/sj.bjc.6604503 PMC2527786

[11] Heng B, Glenn WK, Ye Y, Tran B, Delprado W, et al (2009) Human papilloma virus is associated with breast cancer. BrJCancer 101: 1345–1350. 10.1038/sj.bjc.6605282 PMC2737128 19724278

[12] Kroupis C, Markou A, Vourlidis N, Dionyssiou-Asteriou A, Lianidou ES (2006) Presence of high-risk human papillomavirus sequences in breast cancer tissues and association with histopathological characteristics. ClinBiochem 39: 727–731. 10.1016/j.clinbiochem.2006.03.005 16780823

[13] Gopalkrishna V, Singh UR, Sodhani P, Sharma JK, Hedau ST, et al (1996) Absence of human papillomavirus DNA in breast cancer as revealed by polymerase chain reaction. Breast Cancer ResTreat 39: 197–202. 10.1007/BF01806186 8872328

[14] Lindel K, Forster A, Altermatt HJ, Greiner R, Gruber G (2007) Breast cancer and human papillomavirus (HPV) infection: no evidence of a viral etiology in a group of Swiss women. Breast 16: 172–177. 17088061 10.1016/j.breast.2006.09.001

[15] Bonnet M, Guinebretiere JM, Kremmer E, Grunewald V, Benhamou E, et al (1999) Detection of Epstein-Barr virus in invasive breast cancers. J Natl Cancer Inst 91: 1376–1381. 10451442 10.1093/jnci/91.16.1376

[16] Fina F, Romain S, Ouafik L, Palmari J, Ben AF, et al (2001) Frequency and genome load of Epstein-Barr virus in 509 breast cancers from different geographical areas. Br J Cancer 84: 783–790. 11259092 10.1054/bjoc.2000.1672 PMC2363823

[17] Luqmani Y, Shousha S (1995) Presence of epstein-barr-virus in breast-carcinoma. Int J Oncol 6: 899–903. 21556618 10.3892/ijo.6.4.899

[18] McCall SA, Lichy JH, Bijwaard KE, Aguilera NS, Chu WS, et al (2001) Epstein-Barr virus detection in ductal carcinoma of the breast. J Natl Cancer Inst 93: 148–150. 11208885 10.1093/jnci/93.2.148

[19] Glaser SL, Ambinder RF, DiGiuseppe JA, Horn-Ross PL, Hsu JL (1998) Absence of Epstein-Barr virus EBER-1 transcripts in an epidemiologically diverse group of breast cancers. Int J Cancer 75: 555–558. 9466655 10.1002/(sici)1097-0215(19980209)75:4<555::aid-ijc10>3.0.co;2-8

[20] Lespagnard L, Cochaux P, Larsimont D, Degeyter M, Velu T, et al (1995) Absence of Epstein-Barr virus in medullary carcinoma of the breast as demonstrated by immunophenotyping, in situ hybridization and polymerase chain reaction. Am J Clin Pathol 103: 449–452. 7726142 10.1093/ajcp/103.4.449

[21] Pittet B, Montandon D, Pittet D (2005) Infection in breast implants. The Lancet Infectious Diseases 5: 94–106. 15680779 10.1016/S1473-3099(05)01281-8

[22] Hunt KM, Foster JA, Forney LJ, Schutte UME, Beck DL, et al (2011) Characterization of the Diversity and Temporal Stability of Bacterial Communities in Human Milk. PLoS ONE 6: e21313. 21695057 10.1371/journal.pone.0021313 PMC3117882

[23] Cabrera-Rubio R, Collado MC, Laitinen K, Salminen S, Isolauri E, et al (2012) The human milk microbiome changes over lactation and is shaped by maternal weight and mode of delivery. The American Journal of Clinical Nutrition 96: 544–551. 22836031 10.3945/ajcn.112.037382

[24] Caporaso JG, Kuczynski J, Stombaugh J, Bittinger K, Bushman FD, et al (2010) QIIME allows analysis of high-throughput community sequencing data. Nat Meth 7: 335–336. 10.1038/nmeth.f.303 PMC3156573 20383131

[25] DeSantis TZ, Hugenholtz P, Larsen N, Rojas M, Brodie EL, et al (2006) Greengenes, a Chimera-Checked 16S rRNA Gene Database and Workbench Compatible with ARB. Applied and Environmental Microbiology 72: 5069–5072. 16820507 10.1128/AEM.03006-05 PMC1489311

[26] Edgar RC (2010) Search and clustering orders of magnitude faster than BLAST. Bioinformatics 26: 2460–2461. 20709691 10.1093/bioinformatics/btq461

[27] Schloss PD, Westcott SL, Ryabin T, Hall JR, Hartmann M, et al (2009) Introducing mothur: Open-Source, Platform-Independent, Community-Supported Software for Describing and Comparing Microbial Communities. Applied and Environmental Microbiology 75: 7537–7541. 19801464 10.1128/AEM.01541-09 PMC2786419

[28] Castillo M, Martin-Orue SM, Manzanilla EG, Badiola I, Martin M, et al (2006) Quantification of total bacteria, enterobacteria and lactobacilli populations in pig digesta by real-time PCR. Veterinary Microbiology 114: 165–170. 16384658 10.1016/j.vetmic.2005.11.055

[29] Lin S-Y, Shen F-T, Lai W-A, Zhu Z-L, Chen W-M, et al (2011) Sphingomonas formosensis sp. nov., a polycyclic aromatic hydrocarbon-degrading bacterium isolated from agricultural soil. International Journal of Systematic and Evolutionary Microbiology 62: 1581–1586. 21873513 10.1099/ijs.0.034728-0

[30] Nishio T, Yoshikura T, Itoh H (1997) Detection of Methylobacterium species by 16S rRNA gene-targeted PCR. Applied and Environmental Microbiology 63: 1594–1597. 9097454 10.1128/aem.63.4.1594-1597.1997 PMC168451

[31] Kinjo Y, Wu D, Kim G, Xing G-W, Poles MA, et al (2005) Recognition of bacterial glycosphingolipids by natural killer T cells. Nature 434: 520–525. 15791257 10.1038/nature03407

[32] Siman JH, Forsgren A, Berglund G, Floren CH (1997) Association between Helicobacter pylori and gastric carcinoma in the city of Malmo, Sweden. A prospective study. Scand J Gastroenterol 32: 1215–1221. 9438319 10.3109/00365529709028150

[33] Uemura N, Okamoto S, Yamamoto S, Matsumura N, Yamaguchi S, et al (2001) Helicobacter pylori infection and the development of gastric cancer. N Engl J Med 345: 784–789. 11556297 10.1056/NEJMoa001999

[34] Arthur JC, Perez-Chanona E, Muhlbauer M, Tomkovich S, Uronis JM, et al (2012) Intestinal Inflammation Targets Cancer-Inducing Activity of the Microbiota. Science 338: 120–123. 22903521 10.1126/science.1224820 PMC3645302

[35] Turnbaugh PJ, Ridaura VK, Faith JJ, Rey FE, Knight R, et al (2009) The Effect of Diet on the Human Gut Microbiome: A Metagenomic Analysis in Humanized Gnotobiotic Mice. Science Translational Medicine 1: 6ra14. 10.1126/scitranslmed.3000322 PMC2894525 20368178

[36] Terabe M, Berzofsky JA (2007) NKT cells in immunoregulation of tumor immunity: a new immunoregulatory axis. Trends Immunol 28: 491–496. 17964217 10.1016/j.it.2007.05.008

[37] Hix LM, Shi YH, Brutkiewicz RR, Stein PL, Wang C-R, et al (2011) CD1d-Expressing Breast Cancer Cells Modulate NKT Cell-Mediated Antitumor Immunity in a Murine Model of Breast Cancer Metastasis. PLoS ONE 6: e20702. 21695190 10.1371/journal.pone.0020702 PMC3113806

[38] Naik S, Bouladoux N, Wilhelm C, Molloy MJ, Salcedo R, et al (2012) Compartmentalized Control of Skin Immunity by Resident Commensals. Science 337: 1115–1119. 22837383 10.1126/science.1225152 PMC3513834

[39] Rossini A, Rumio C, Sfondrini L, Tagliabue E, Morelli D, et al (2006) Influence of Antibiotic Treatment on Breast Carcinoma Development in Proto-neu Transgenic Mice. Cancer Research 66: 6219–6224. 16778196 10.1158/0008-5472.CAN-05-4592

[40] Velicer CM, Heckbert SR, Lampe JW, Potter JD, Robertson CA, et al (2004) Antibiotic use in relation to the risk of breast cancer. JAMA 291: 827–835. 14970061 10.1001/jama.291.7.827

