# Supplementary figures and images for: Microbial Dysbiosis Is Associated with Human Breast Cancer
Source: PLoS One. 2014 Jan 8;9(1):e83744. doi: 10.1371/journal.pone.0083744 (PMC3885448; doi:10.1371/journal.pone.0083744)

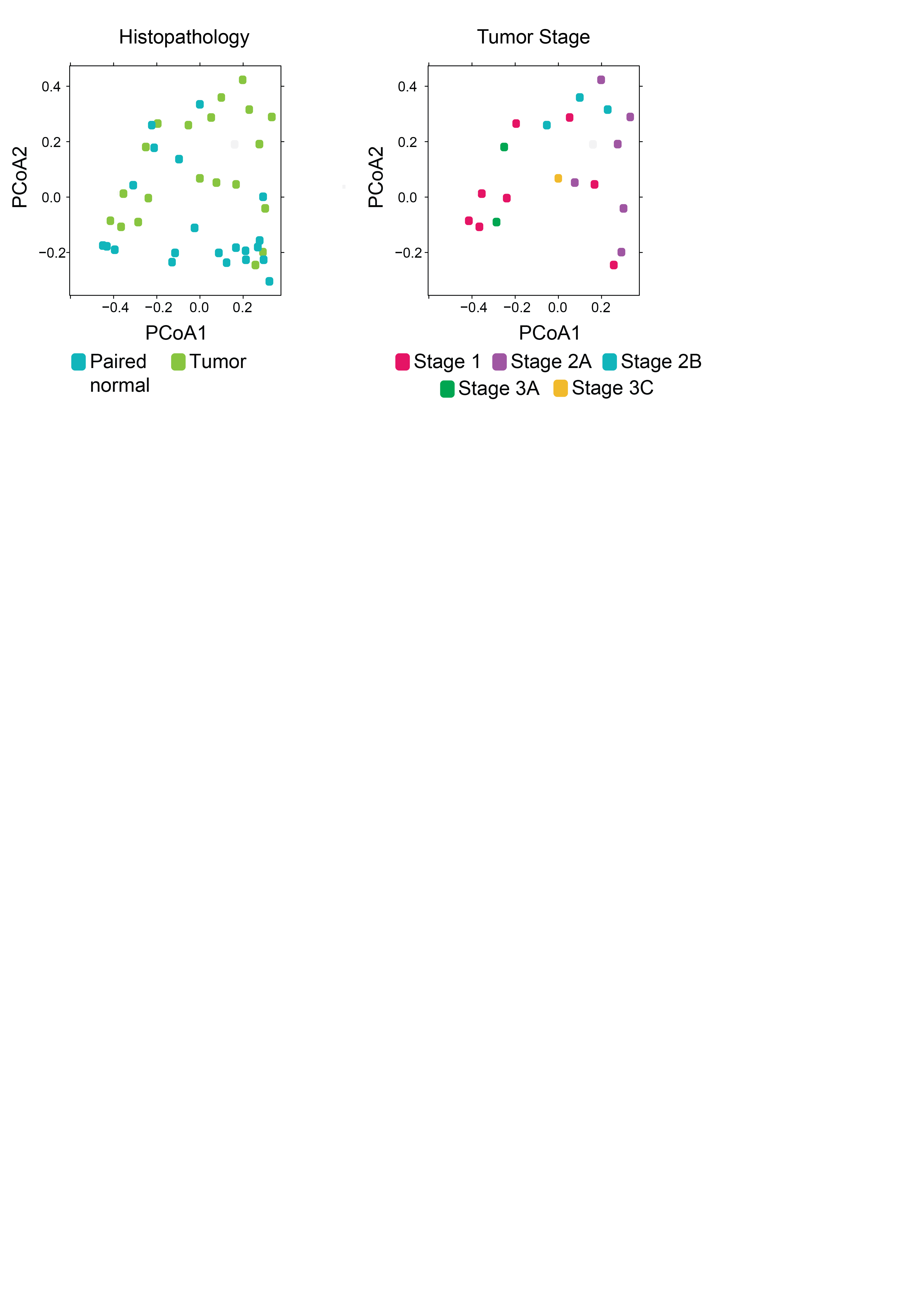

Supplement: Figure S1 — Principle coordinates analysis (PCoA) plots of samples categorized based on histopathology (left panel, n = 20 paired samples) or tumor stage (right panel, n = 20 tumor only). No clustering based on these categories was found among samples. (TIF) [file pone.0083744.s001.tif]

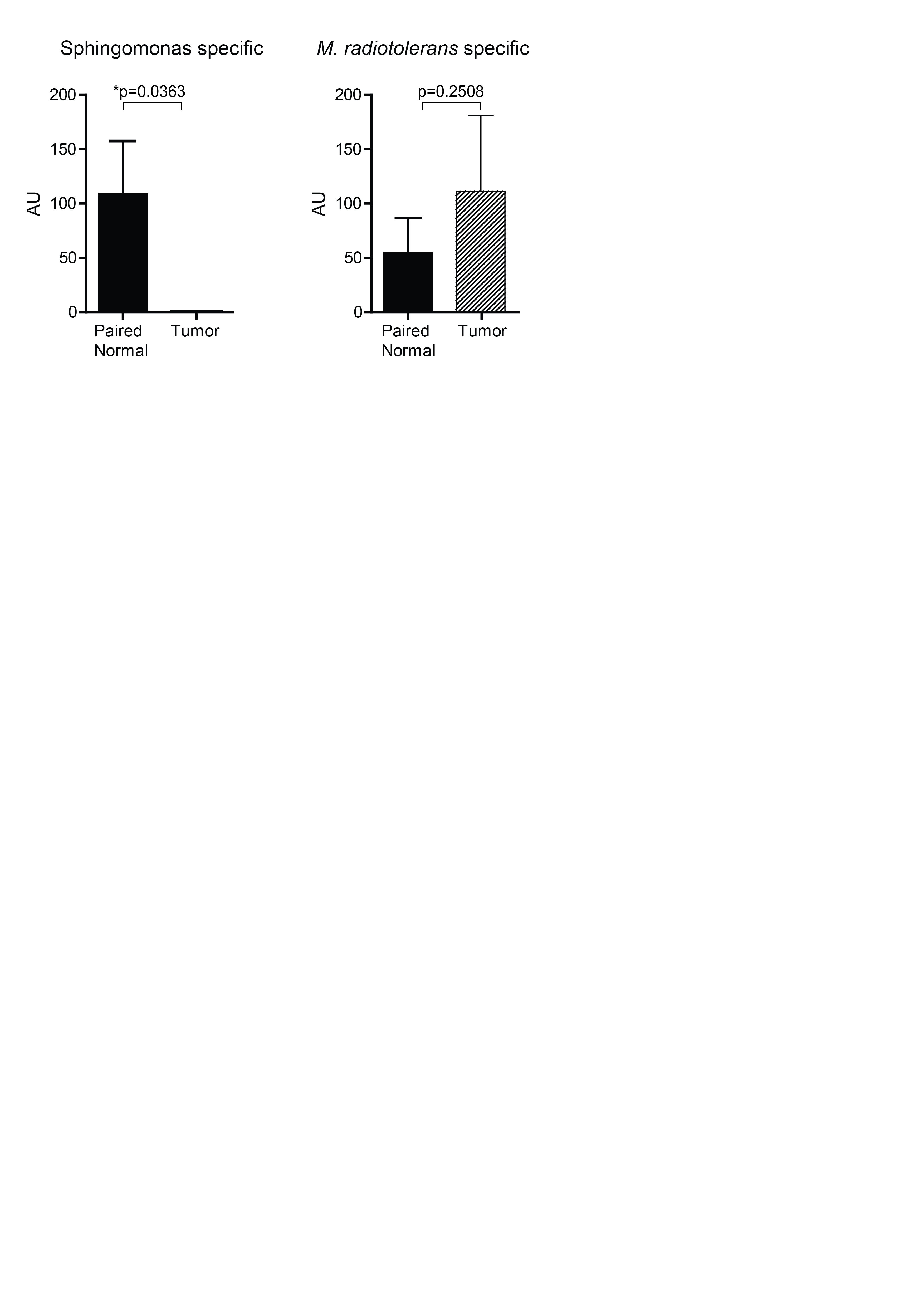

Supplement: Figure S2 — Detection of Sphingomonas and M. radiotolerans in paired normal and breast tumor tissues (n = 20). Data represent the average of duplicate values. Data were normalized to expression levels of beta-actin. p-values from Student’s paired t-test are shown, with p<0.05 considered significant. Error bars represent mean ± s.e.m. (TIF) [file pone.0083744.s002.tif]

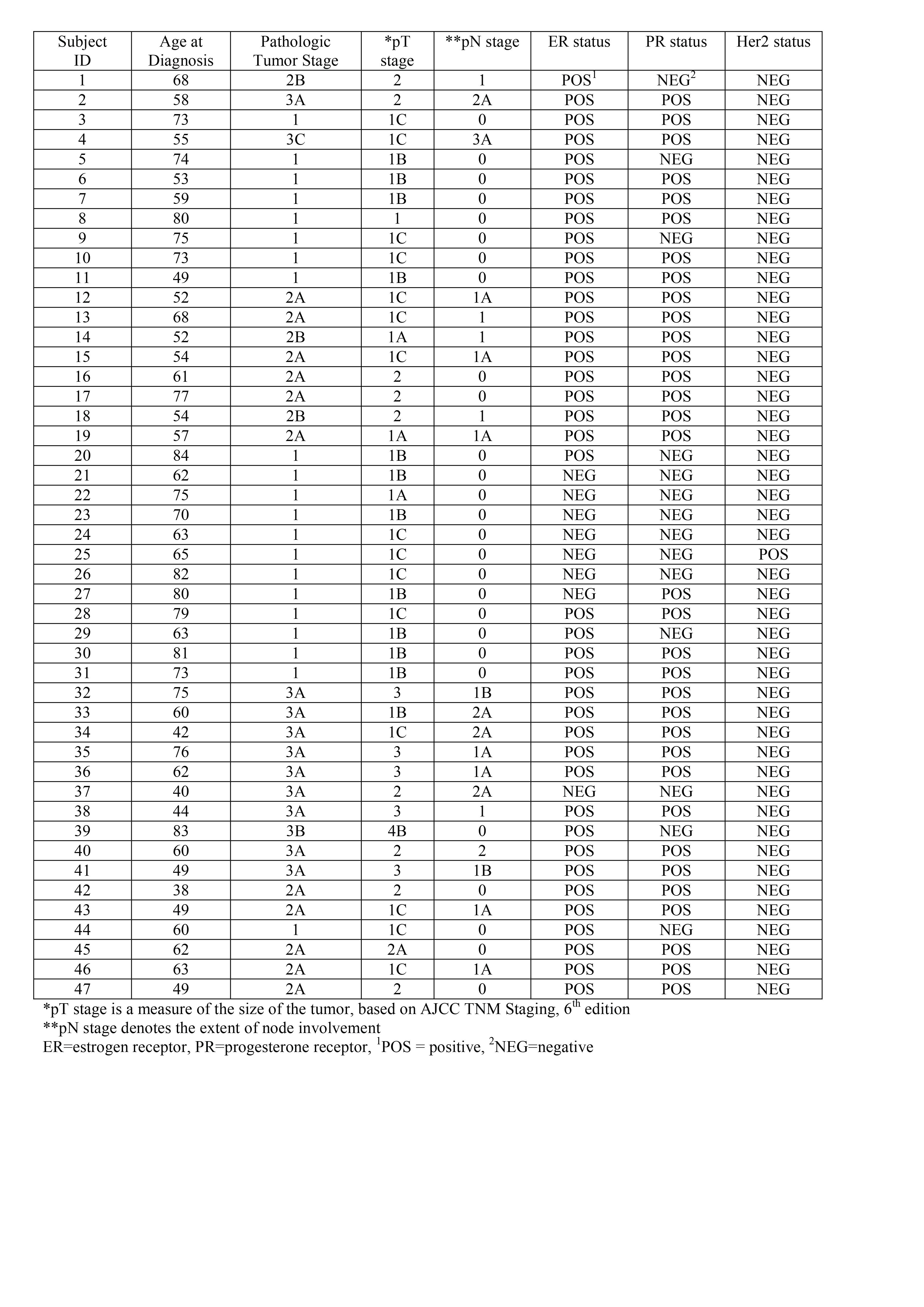

Supplement: Table S1 — Summary of clinical data for the breast cancer patients used in this study. (TIF) [file pone.0083744.s003.tif]

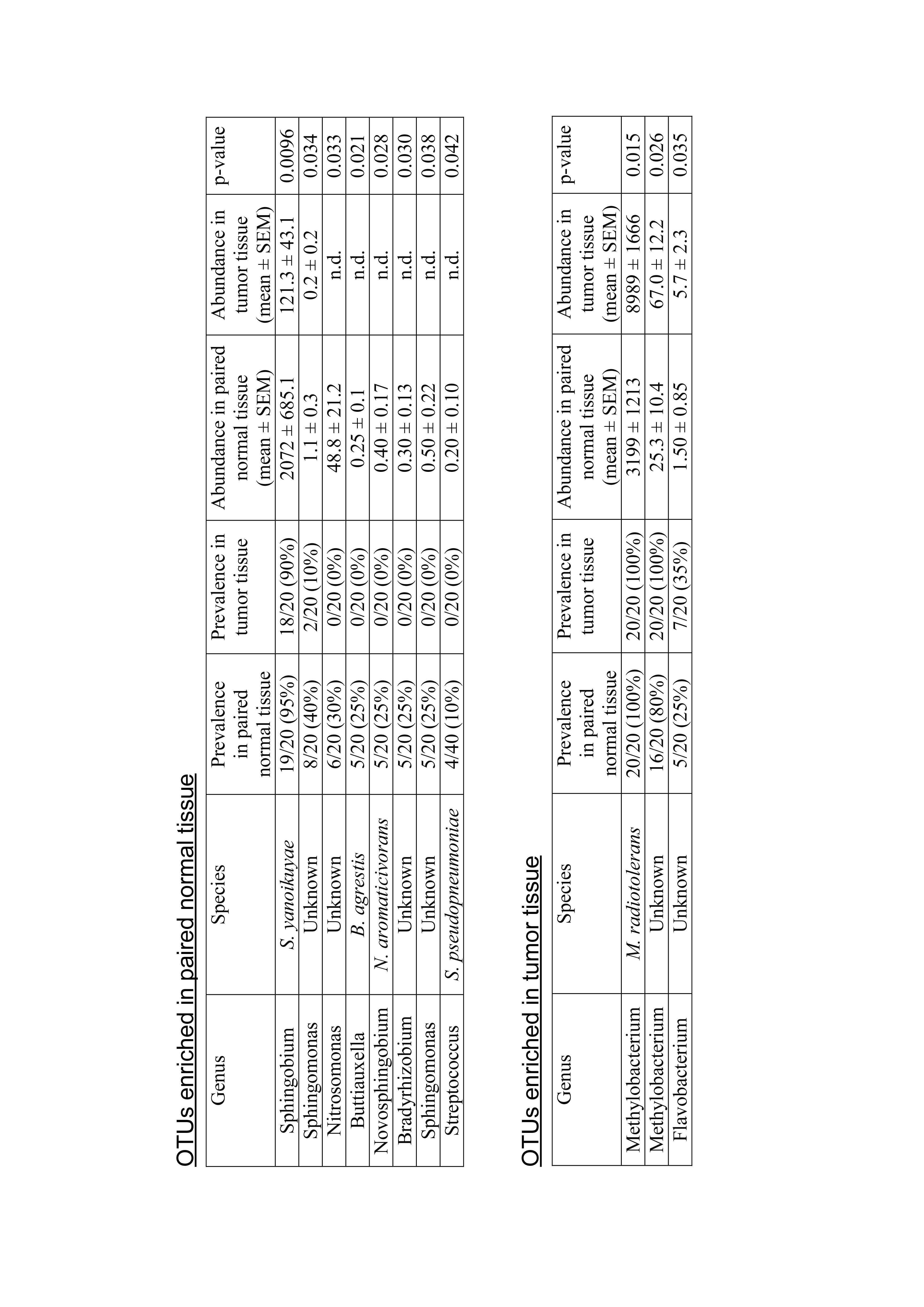

Supplement: Table S2 — OTUs enriched in paired normal or tumor tissue. Prevalence refers to the number of samples in which the indicated OTU was detectable. Paired Student’s t-tests were used to determine differences in abundances of OTUs. n.d., not detectable. (TIF) [file pone.0083744.s004.tif]
